# Supplementary material for: Pseudomonas aeruginosa Uses c-di-GMP Phosphodiesterases RmcA and MorA To Regulate Biofilm Maintenance
Source: mBio. 2021 Feb 2;12(1):e03384-20. doi: 10.1128/mBio.03384-20 (PMC7858071; doi:10.1128/mBio.03384-20)
Supplement: FIG S2 [file mBio.03384-20-sf002.pdf]

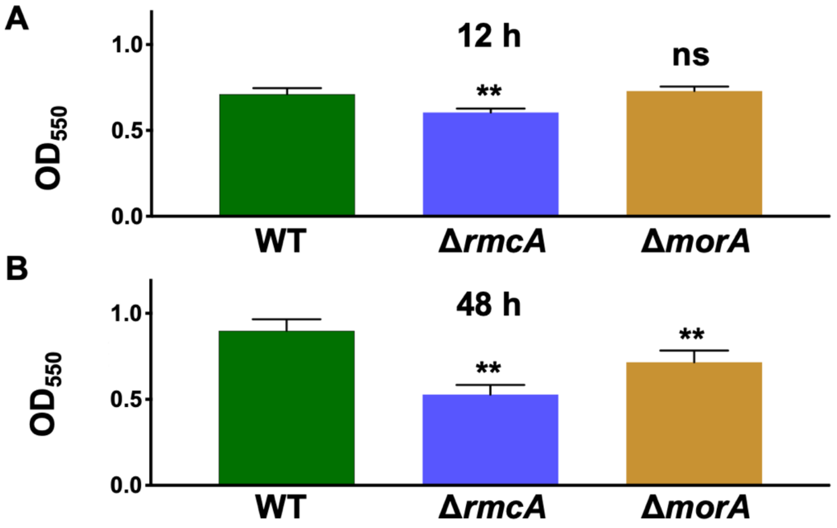

Figure S2. The Δ*rmcA* and Δ*morA* mutations exhibit biofilm maintenance defect in the *P. aeruginosa* PAO1 background. Biofilms were grown in M63 minimal medium supplemented with 0.4% for 12 h (panel A) and 48 h (panel B), representing early and late stages of biofilm formation, respectively. Error bars represent standard deviation of the results from three biological replicates each performed with three technical replicates and tested for significance using an unpaired Student's T-test. **indicate a difference in biofilm that is significantly different at a P value of <0.01, 0.001 and 0.0001, respectively, compared to WT.
