# Supplementary material for: Pseudomonas aeruginosa Uses c-di-GMP Phosphodiesterases RmcA and MorA To Regulate Biofilm Maintenance
Source: mBio. 2021 Feb 2;12(1):e03384-20. doi: 10.1128/mBio.03384-20 (PMC7858071; doi:10.1128/mBio.03384-20)
Supplement: FIG S3 [file mBio.03384-20-sf003.pdf]

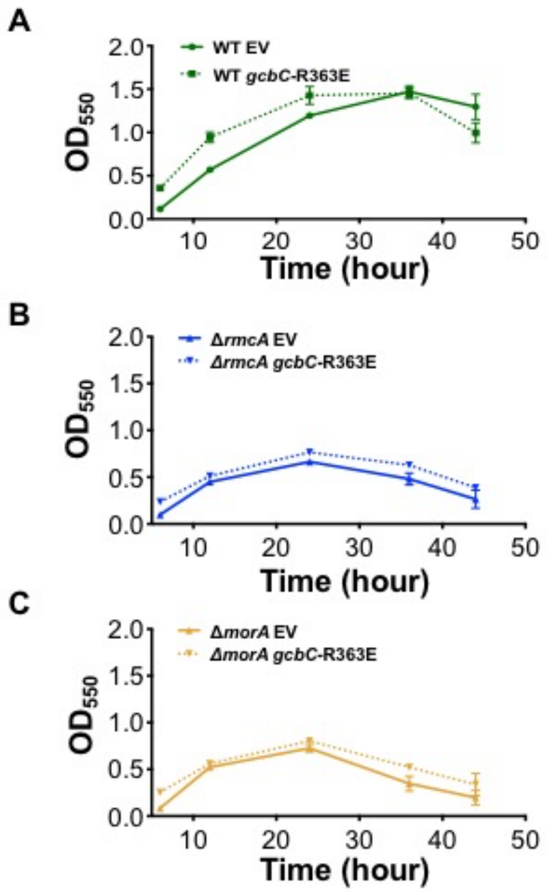


**Figure S3. Elevated c-di-GMP does not in increase the biofilm deficit in the Δ*morA* or Δ*rmcA* mutants**. Static biofilms of (A) WT, and the (B) Δ*rmcA* and (C) Δ*morA* mutants with either the empty vector (EV, solid line) or a plasmid carrying a gene expressing the constitutively active GcbC-R363E protein, which over-produces c-di-GMP (dotted line), were grown in M63 medium with 0.4% L-arginine plus 0.2% arabinose to induce expression of the mutant protein, and biofilm growth measured at the indicate time points. Results are representative of three biological replicates each with three technical replicates. There were no significant differences at any time point.
