# Supplementary material for: Pseudomonas aeruginosa Uses c-di-GMP Phosphodiesterases RmcA and MorA To Regulate Biofilm Maintenance
Source: mBio. 2021 Feb 2;12(1):e03384-20. doi: 10.1128/mBio.03384-20 (PMC7858071; doi:10.1128/mBio.03384-20)
Supplement: FIG S4 [file mBio.03384-20-sf004.pdf]

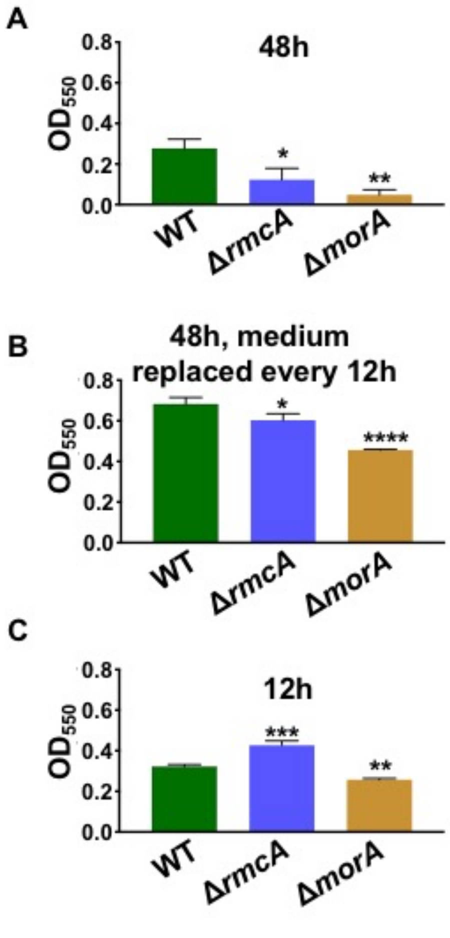

Figure S4. The biofilm maintenance defect can be partially rescued in the static assay with fresh medium. (A) Biofilms were grown in M63 minimal medium supplemented with 0.4% arginine and grown for 48 h to a late stage of biofilm formation. (B) To alleviate nutrient limitation conditions, the medium was replaced every 12 h and biofilm measured after 48 h growth (i.e., four medium replacements). (C) Biofilms were grown in M63 minimal medium supplemented with 0.4% arginine and grown for 12 h as a control for early stage biofilm formation. Error bars represent standard deviation of the results from three biological replicates each performed with three technical replicates and tested for significance using an unpaired Student's T-test. *,**, ***, **** indicate a difference in biofilm that is significantly different at a P value of <0.05, 0.01, 0.001 and 0.0001, respectively, compared to the WT.
