# Supplementary material for: Pseudomonas aeruginosa Uses c-di-GMP Phosphodiesterases RmcA and MorA To Regulate Biofilm Maintenance
Source: mBio. 2021 Feb 2;12(1):e03384-20. doi: 10.1128/mBio.03384-20 (PMC7858071; doi:10.1128/mBio.03384-20)
Supplement: FIG S5 [file mBio.03384-20-sf005.pdf]

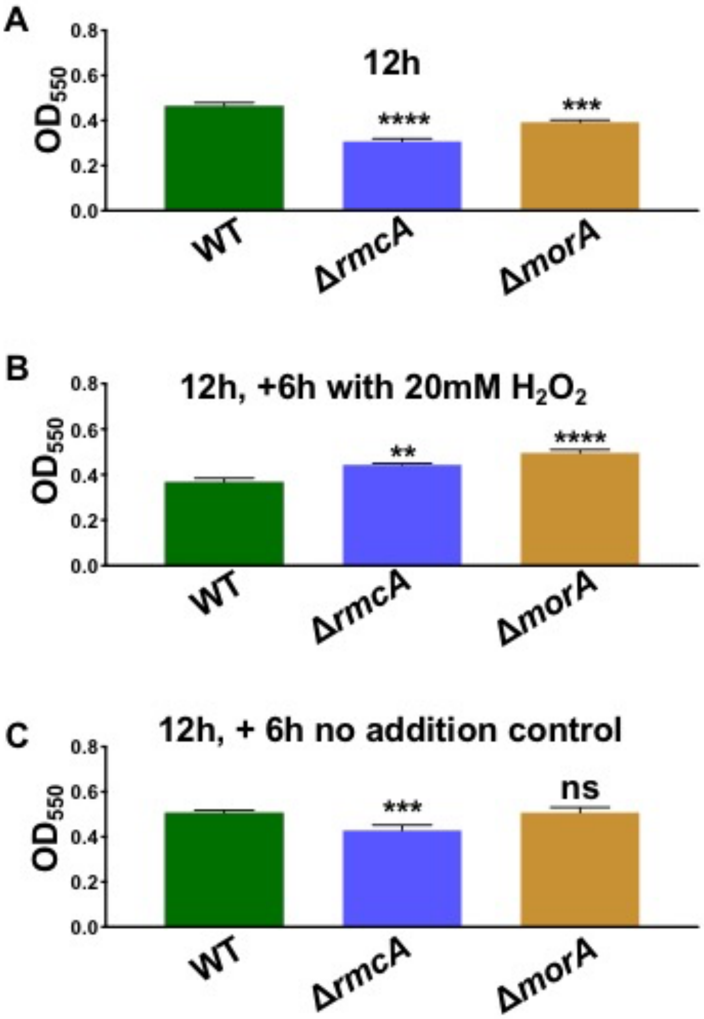

Figure S5. Peroxide addition does not induce a biofilm maintenance defect. Biofilms were grown statically in M63 minimal medium supplemented with 0.4% arginine and measured at 12 h when nutrient limitation does not predominate (A), after an additional 6 hours of growth in medium containing 20 mM H_2O_2 (B) and compared with the biofilm formed after additional 6 hours of growth in medium with no added H_2O_2 (C). Error bars represent standard deviation of the results from three biological replicates each performed with three technical replicates and tested for significance using an unpaired Student's T-test. **, ***, **** indicate a difference in biofilm that is significantly different at a P value of <0.01, 0.001 and 0.0001, respectively, compared to the WT.
