# Supplementary material for: Pseudomonas aeruginosa Uses c-di-GMP Phosphodiesterases RmcA and MorA To Regulate Biofilm Maintenance
Source: mBio. 2021 Feb 2;12(1):e03384-20. doi: 10.1128/mBio.03384-20 (PMC7858071; doi:10.1128/mBio.03384-20)
Supplement: FIG S6 [file mBio.03384-20-sf006.pdf]

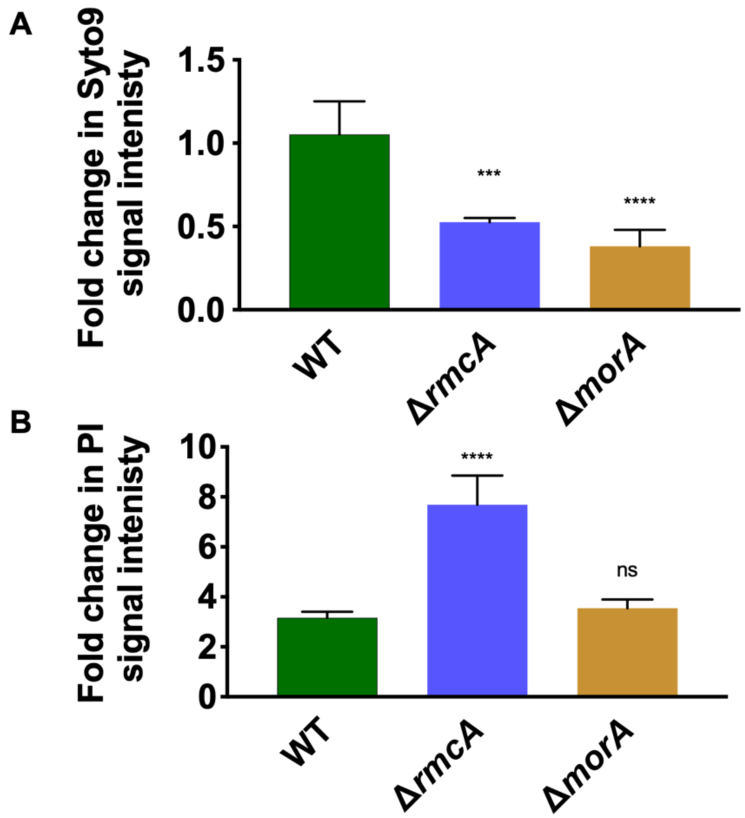

Figure S6. Cell death during late-stage biofilm differs between Δ*rmcA* and Δ*morA* mutants. Data from Figure 3B was assessed for the change in fluorescent intensity observed in late (48 h) versus early (16 h) biofilms of Syto9- (A) and PI-stained (B) biofilms. Error bars represent standard deviation from three technical replicates and are representative of experimental results observed over four biological replicates and tested for significance using an unpaired Student's T-test. ***, **** indicate a difference in biofilm that is significantly different at a P value of <0.001 or 0.0001, respectively, compared to the WT. ns indicates a non-significant difference.
