# Supplementary material for: Pseudomonas aeruginosa Uses c-di-GMP Phosphodiesterases RmcA and MorA To Regulate Biofilm Maintenance
Source: mBio. 2021 Feb 2;12(1):e03384-20. doi: 10.1128/mBio.03384-20 (PMC7858071; doi:10.1128/mBio.03384-20)
Supplement: FIG S7 [file mBio.03384-20-sf007.pdf]

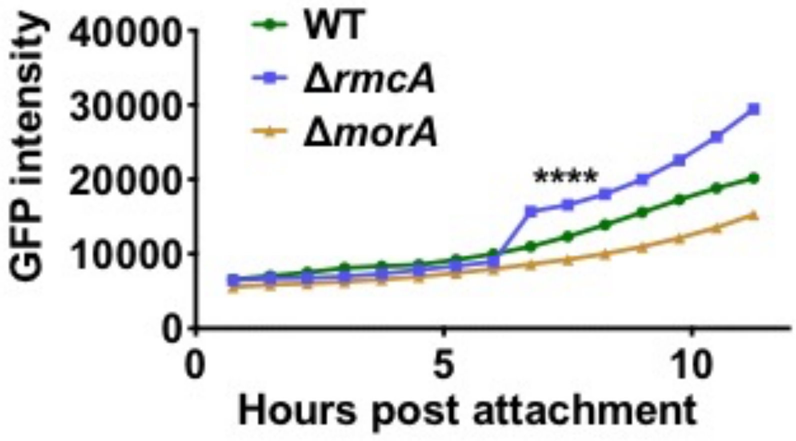

Figure S7. **Initial biofilm formation by the WT and Δ*rmcA* and Δ*morA* mutants**. The WT strain and the Δ*rmcA* and Δ*morA* mutants carrying pSMC21, a plasmid which constitutively expresses GFP, were inoculated into microfluidic chambers and allowed 1 hour to attach prior to beginning flow of the medium. GFP fluorescence was measured every 45 minutes for 12 h, plotted and tested for significance using a one-way multiple comparisons ANOVA. **** indicate a difference in biofilm that is significantly different from the WT at a P value of <0.0001 at all times after 7 h of growth. There was no significant difference between WT and the *morA* mutant.
