# Supplementary material for: Pseudomonas aeruginosa Uses c-di-GMP Phosphodiesterases RmcA and MorA To Regulate Biofilm Maintenance
Source: mBio. 2021 Feb 2;12(1):e03384-20. doi: 10.1128/mBio.03384-20 (PMC7858071; doi:10.1128/mBio.03384-20)
Supplement: FIG S8 [file mBio.03384-20-sf008.pdf]

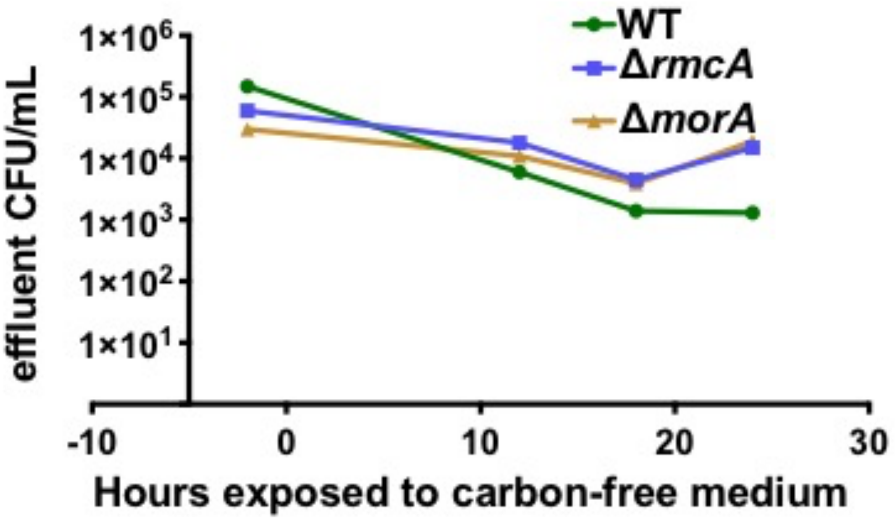

Figure S8. Viable count of the WT and PDE mutants in the microfluidic device effluent. Bacteria were grown under flow in biofilm medium containing 0.4% arginine for 24 h and exposed to nutrient-limited (arginine-free) medium for an additional 24 h for starvation response to occur. CFUs in effluent 2 h prior and 12, 18 and 24 h after biofilms were exposed to nutrient-limited conditions were captured and grown on LB plates. Results shown are the average of two biological replicates.
