# Supplementary material for: Pseudomonas aeruginosa Uses c-di-GMP Phosphodiesterases RmcA and MorA To Regulate Biofilm Maintenance
Source: mBio. 2021 Feb 2;12(1):e03384-20. doi: 10.1128/mBio.03384-20 (PMC7858071; doi:10.1128/mBio.03384-20)
Supplement: FIG S9 [file mBio.03384-20-sf009.pdf]

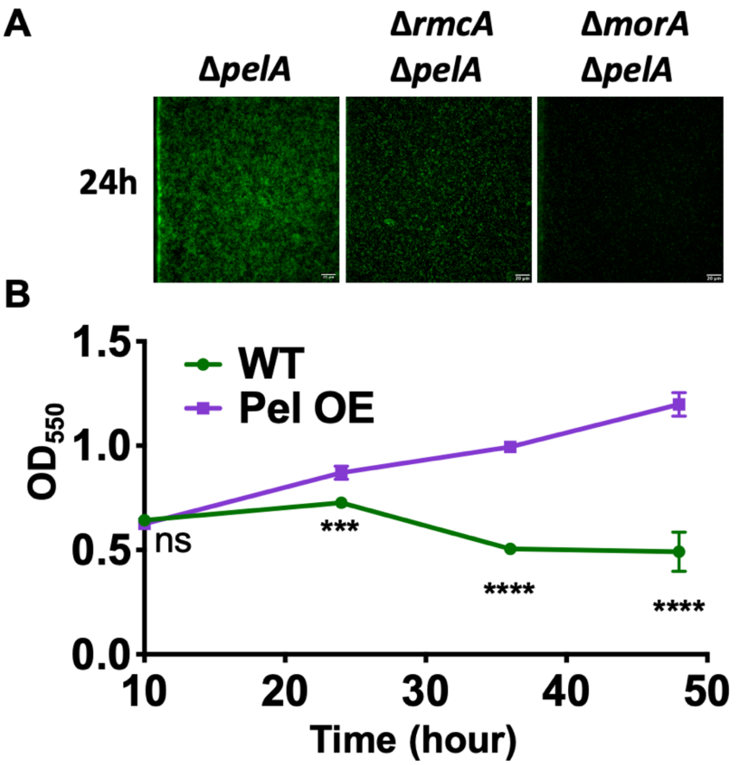

Figure S9. Late stage biofilm defect cannot be induced or rescued with changes to *pel* expression alone. (A) Overnight cultures of Δ*pelA*, Δ*rmcA* Δ*pelA* and Δ*rmcA* Δ*pelA* mutants strains were inoculated into a microfluidics chamber and assayed for biofilm development at 24 h. (B) WT and an inducible Pel strain under the control of the P_{BAD} promoter were compared for the presence of a late-stage biofilm defect. The Y-axis shows a measure of the biofilm formed. Biofilms were grown statically in KA biofilm medium supplemented with 0.4% arginine for 10, 24, 36 and 48 h, plotted and tested for significance using a one-way multiple comparisons ANOVA. Pel production was induced by the addition of 0.2% arabinose. ***, **** indicate a difference in biofilm that is significantly different at a P value of <0.001 and 0.0001, respectively, compared to the WT. NS indicates a non-significant result. Results shown are representative of two biological replicates each containing three technical replicates.
