# Supplementary material for: Pseudomonas aeruginosa Uses c-di-GMP Phosphodiesterases RmcA and MorA To Regulate Biofilm Maintenance
Source: mBio. 2021 Feb 2;12(1):e03384-20. doi: 10.1128/mBio.03384-20 (PMC7858071; doi:10.1128/mBio.03384-20)
Supplement: TABLE S1 [file mBio.03384-20-st001.pdf]

Table S1. Bacterial strains, plasmids and primers used in this study.

Strain	Description	Source
E. coli		
DH5α	Cloning strain; F⁻ Φ80*lacZ*ΔM15 Δ(*lacZYA-argF*) U169 *recA1 endA1 hsdR17* (r_K^-, m_K^+) *phoA supE44* λ⁻ *thi*-1 *gyrA96 relA1*	Invitrogen
SM10	Bi-parental mating strain; *thi thr leu tonA lacY supE recA*::RP4-2-Tc::Mu K_m λ*pir*, Kan^R, Tet^R	(1)
BTH101	BACTH strain; F⁻ *cya-99 araD139 galE15 galK16 rpsL1* (Str^R) *hsdR2 mcrA1 mcrB1*	Euromedex
P. aeruginosa		
PAO1	Wild-type strain	M. Parsek
PAO1 Δ*wspF* Δ*psl* P_{BAD}*pel*	PAO1 Δ*wspF* (in-frame); Δ*pslBCD* (polar); *araC*-P_{BAD} inserted upstream of *pelABCDEFG*	(2)
GBW1	PAO1 Δ*wspF* Δ*psl* P_{BAD}*pel* Δ*rmcA*	This study
GBW2	PAO1 Δ*wspF* Δ*psl* P_{BAD}*pel* Δ*morA*	This study
GBW3	PAO1 Δ*wspF* Δ*psl* P_{BAD}*pel* Δ*rmcA attTn7*::miniTn7T-Gm::*araC*-P_{BAD}::*rmcA*, Gen^R	This study
GBW4	PAO1 Δ*wspF* Δ*psl* P_{BAD}*pel* Δ*morA attTn7*::miniTn7T-Gm::*araC*-P_{BAD}::*morA*, Gen^R	This study
GBW5	PAO1 Δ*wspF* Δ*psl* P_{BAD}*pel* Δ*rmcA attTn7*::miniTn7T-Gm::*araC*-P_{BAD}::*rmcA*$^{C\text{-}VSV\text{-}G}$, Gen^R	This study
GBW6	PAO1 Δ*wspF* Δ*psl* P_{BAD}*pel* Δ*morA attTn7*::miniTn7T-Gm::*araC*-P_{BAD}::*morA*$^{C\text{-}VSV\text{-}G}$, Gen^R	This study
SMC232	UCBPP-PA14	(3)

SMC6279	PA14 Δ*rmcA* (in-frame)	(4)
SMC6289	PA14 Δ*morA* (in-frame)	(4)
SMC7006	PA14 *att*::$DMS3_{42}$	(5)
SMC2893	PelA KO	(6, 7)
SMC6607	Pel inducible, P_{pel} replaced with P_{bad}	(8)
SMC8808	PA14 Δ*rmcA att*::$DMS3_{42}$	This study
SMC8809	PA14 Δ*morA att*::$DMS3_{42}$	This study
SMC8810	PA14 pKNT25 GcbC R363E	This study
SMC8811	PA14 Δ*rmcA* pKNT25 GcbC R363E	This study
SMC8812	PA14 Δ*morA* pKNT25 GcbC R363E	This study
SMC8813	PA14 Δ*rmcA attTn7*::miniTn7T-Gm::*araC*-P_{BAD}::*rmcA*, Gen^R	This study
SMC8814	PA14 Δ*morA attTn7*::miniTn7T-Gm::*araC*-P_{BAD}::*morA*, Gen^R	This study
SMC8815	PA14 Δ*rmcA*:P_{tac}-*mKO*-κ	This study
SMC8816	PA14 Δ*morA*:P_{tac}-*mKO*-κ	This study
SMC8817	PA14 Δ*rmcA*:Psmc21-*gfp*	This study
SMC8818	PA14 Δ*morA*:Psmc21-*gfp*	This study
SMC8819	PA14 Δ*rmcA* Δ*pelA*	This study
SMC8820	PA14 Δ*morA* Δ*pelA*	This study
SMC8821	PA14 Δ*rmcA,* inducible Pel under the control of P_{BAD}	This study
SMC8822	PA14 Δ*morA* inducible Pel under the control of P_{BAD}	This study

Plasmid	Description	Source
**Two-step allelic exchange**		
pEX18Gm	Suicide vector for allelic exchange in *P. aeruginosa*, encodes SacB, Gen^R	(1)
pEX18Gm::Δ*rmcA*	pEX18Gm with *P. aeruginosa* PAO1 Δ*rmcA* allele cloned between the EcoRI and HindIII sites, Gen^R	This study
pEX18Gm::Δ*morA*	pEX18Gm with *P. aeruginosa* PAO1 Δ*morA* allele cloned between the SacI and HindIII sites, Gen^R	This study
pSMC21	pUCP-based plasmid containing gfpmut2, Ap^r , Cb^r , Km^r	(9)
pMQ30	Suicide vector; Gm^r, *sacB*, *URA3*, *CEN6/ARSH4*, *lacZα*	(10)
pKO PA14_07500	PA14_07500 knockout construct in pMQ30	(4)
pKO PA14_07500	PA14_60870 (*morA*) knockout construct in pMQ30	(4)
pKNT25 GcbC-R363E	GcbC protein with an I-site mutation	(11)
**Complementation analysis**		
pUC18T-miniTn7T-Gm	*aacC1* on miniTn7-based vector with transcriptional terminators at the right end of the Tn7 transposon; Amp^R, Gen^R	(12)
pUC18T-miniTn7T-Gm-pBAD	pUC18T-miniTn7T-Gm containing *araC*-P_{BAD} and a downstream MCS (SmaI-NotI-PstI-NcoI) cloned between the HindIII and SacI sites; Amp^R, Gen^R	This study

pTNS2	Helper plasmid encoding *tnsABCD*, Amp^R	(13)
p-miniTn7-*rmcA*	pUC18T-miniTn7T-Gm-pBAD with *P. aeruginosa* PAO1 *rmcA* fused to an upstream synthetic ribosome binding site, cloned between the SmaI and NotI sites; Amp^R, Gen^R	This study
p-miniTn7-*morA*	pUC18T-miniTn7T-Gm-pBAD with *P. aeruginosa* PAO1 *morA* fused to an upstream synthetic ribosome binding site, cloned between the NotI and Mph1103I sites; Amp^R, Gen^R	This study
p-miniTn7-*rmcA*$^{C\text{-}VSV\text{-}G}$	p-miniTn7-*rmcA* fused to a downstream VSV-G tag; Amp^R, Gen^R	This study
p-miniTn7-*morA*$^{C\text{-}VSV\text{-}G}$	p-miniTn7-*morA* fused to a downstream VSV-G tag; Amp^R, Gen^R	This study
**BACTH assays**		
pUT18C	Encodes T18 fragment of *B. pertussis* adenylate cyclase toxin on N-terminus, Amp^R	(14)
pKT25	Encodes T25 fragment of *B. pertussis* adenylate cyclase toxin on N-terminus, Kan^R	(14)
pUT18C::zip	BACTH positive control, pUT18C containing sequence coding for the leucine zipper region of yeast GCN4 protein, Amp^R	(14)
pKT25::zip	BACTH positive control, pKT25 containing sequence coding for the leucine zipper region of yeast GCN4 protein, Kan^R	(14)

pKT25::*pelD*	*P. aeruginosa* PAO1 *pelD* cloned into the XbaI and BamHI sites of pKT25, Kan^R	(15)
pUT18C::*rmcA*	*P. aeruginosa* PAO1 *rmcA* cloned into the XbaI and SmaI sites of pUT18C, Amp^R	This study
pUT18C::*morA*	*P. aeruginosa* PAO1 *morA* cloned into the XbaI and KpnI sites of pUT18C, Amp^R	This study
pBADGr	For expression of third protein in the BACTH assay, *ori araC*-P_{BAD} *dhfr*::Gm^R mob^+, Gen^R	(16)
pBADGr::*pelE*	*P. aeruginosa* PAO1 *pelE* cloned into the EcoRI and HindIII sites of pBADGr with synthetic RBS (GAGGAGGATATTC), Gen^R	(15)
pBADGr::*pelG*	*P. aeruginosa* PAO1 *pelG* cloned into the EcoRI and HindIII sites of pBADGr with synthetic RBS (GAGGAGGATATTC), Gen^R	(15)
pBADGr::*pelEG*	*P. aeruginosa* PAO1 *pelE* and *pelG* ORFs separated by a clean, unmarked deletion of *pelF* cloned into the EcoRI and HindIII sites of pBADGr with synthetic RBS (GAGGAGGATATTC), Gen^R	This study

Abbreviations: Amp, ampicillin; Kan, kanamycin; Gen, gentamicin; Str, streptomycin; Tet, tetracycline

Primers	Sequence*
**Allelic exchange vectors**	
*rmcA*PAO1upF	GGG **GAA TTC** GAA CCA CGA GAC CGA CGA GA
*rmcA*PAO1upR	*TAC CGG CGC TTC TCG GGG AGC* ACA CAG GAG AAG CAC GGC CTG
*rmcA*PAO1downF	GCT CCC CGA GAA GCG CCG GTA
*rmcA*PAO1downR	AGG **AAG CTT** GGA ATA ACC AAC GGT GCC AGG T
*morA*PAO1upF	AAT **GAG CTC** TCC TAC CTG GAA GCG CTG GA
*morA*PAO1upR	*TCA GCC CTC GTT GAA CAT GAA* CGA CGG GGT TAG CGA GGG GGT
*morA*PAO1downF	TTC ATG TTC AAC GAG GGC TGA
*morA*PAO1downR	GGG **AAG CTT** AGG TAG ACC GCG GCG TTG GC
**miniTn7 complementation vectors**	
miniTn7-pBAD-F	GGG **AAG CTT** TTA TGA CAA CTT GAC GGC TA
miniTn7-pBAD-R	GGG **GAG CTC** CCA TGG CTG CAG GCG GCC GCC CCG GGC AAA AAA ACG GGT ATG GAG AAA CAG TA
rmcA-miniTn7-F	GAG **CCC GGG *AGG AGG ATA TTC*** ATG CAG CGT CTG CAG GCC GT
rmcA-miniTn7-R	GGG **GCG GCC GC**T TAT ACC GGC GCT TCT CGG GG
morA-miniTn7-F	GGG **GCG GCC GC*A GGA GGA TAT TC***G TGT CGA CCC CCT CGC TAA C
morA-miniTn7-R	GGG **ATG CAT** TCA GCC CTC GTT GAA CAT GAA CA
rmcA-C-VSV-G-F	TGA ATA GAT TAG GAA AAT AAG CGG CCG CCT GCA GC
rmcA-C-VSV-G-R	TTT CAA TAT CTG TAT ATA CCG GCG CTT CTC GGG GA

morA-C-VSV-G-F	ATA GAT TAG GAA AAT GAA TGC ATG ATC GAA TTA GCT TCA A
morA-C-VSV-G-R	TCA TTT CAA TAT CTG TAT AGC CCT CGT TGA ACA TGA ACA G
**BACTH vectors**	
pelD-BACTH-F	GGG **TCT AGA** GAT GTC CGC GCA CAA GGA TTT CA
pelD-BACTH-R	GTT **GGA TCC** CCT AAA TAG CCA CTT GCT GAT CAT TC
rmcA-BACTH-F	GAT **TCT AGA** GAT GCA GCG TCT GCA GGC CG
rmcA-BACTH-R	GAG **CCC GGG** TTA TAC CGG CGC TTC TCG GG
morA-BACTH-F	GAG **TCT AGA** GGT GTC GAC CCC CTC GCT AA
morA-BACTH-R	GTG **GGT ACC** TCA GCC CTC GTT GAA CAT GAA C
pelE-BADGr-F	GGG **GAA TTC** GAG GAG GAT ATT CAT GAT CAG CAA GTG GCT ATT TAG C
pelE-BADGr-R	GAG **AAG CTT** TCA TGT CCA GTA TCT CGC CAG
pelG-BADGr-F	GGG **GAA TTC** GAG GAG GAT ATT CAT GGC CGG CAT CGG CTTC
pelG-BADGr-R	GAG **AAG CTT** TCA GCG ATT GAG CAT GAA GGT
pelF-del-BADGr-upR	*TCA TGC AAT CTC CGT GGC TT*C CGG AGC GGT GTG TTC GGT CAT
pelF-del-BADGr-downF	GAA GCC ACG GAG ATT GCA TGA
**Sequencing primers**	
M13F	GTA AAA CGA CGG CCA G
M13R	CAG GAA ACA GCT ATG AC
rmcA-SEQ-F	TGG ACA TGC CTG AGG ACA AG
rmcA-SEQ-R	CGC CTG TTT CAG CAC GTC AAA A

morA-SEQ-F	CGG ACT TCC TCG ACC CG
morA-SEQ-R	CGC CAT GGG CCA GGC T
miniTn7-SEQ-F	GCG GAT CCT ACC TGA CGC TT
miniTn7-SEQ-R	AAC TTC AGA GCG CTT TTG AA
pUT18C-SEQ-F	CTC GCC GGA TGT ACT GGA AAC
pUT18C-SEQ-R	CGG GGC TGG CTT AAC TAT GC
pKT25-SEQ-F	GGC GCG CAG TTC GGT GAC CAG CGG C
pKT25-SEQ-R	GGG ATG TGC TGC AAG GCG ATT AAG
pBADGr-SEQ-F	ATG CCA TAG CAT TTT TAT CC
pBADGr-SEQ-R	GAT TTA ATC TGT ATC AGG
rmcA-SEQ-int1	GGA GAT CGA GCG ACT CCA GG
rmcA-SEQ-int2	CGA GCA GCA GAT CGG CAT CG
rmcA-SEQ-int3	TCA ACC GCG CCT TCA GCG AG
rmcA-SEQ-int4	ATC GCC CTG TTC CCC AAG GA
morA-SEQ-int1	TGC TCG ACG ACA TGC AGA CC
morA-SEQ-int2	TGC GCC AGG CTC TTG TGG AG
morA-SEQ-int3	TGG CAC TGG TTC GAC ATC CG
morA-SEQ-int4	ACC GCC AGG AAG CCA ACC AG

*Restriction sites and Gateway *att* sequences are bolded; regions of complementary to the target amplicon are underlined; regions of reverse complementarity (to facilitate splicing) are italicized; synthetic ribosomal binding sites are in bold italics

References

1. Hoang TT, Karkhoff-Schweizer RR, Kutchma AJ, Schweizer HP. 1998. A broad-host-range F1p-FRT recombination system for site-specific excision of chromosomally-located DNA sequences: Application for isolation of unmarked *Pseudomonas aeruginosa* mutants. Gene 212:77–86.
2. Colvin KM, Alnabelseya N, Baker P, Whitney JC, Lynne Howell P, Parsek MR. 2013. PelA deacetylase activity is required for *pel* polysaccharide synthesis in *Pseudomonas aeruginosa*. Journal of Bacteriology 195:2329–2339.
3. Rahme LG, Stevens EJ, Wolfort SF, Shao J, Tompkins RG, Ausubel FM. 1995. Common virulence factors for bacterial pathogenicity in plants and animals. Science 268:1899–1902.
4. Ha DG, Richman ME, O'Toole GA. 2014. Deletion mutant library for investigation of functional outputs of cyclic diguanylate metabolism in *Pseudomonas aeruginosa* PA14. Applied and Environmental Microbiology 80:3384–3393.
5. Heussler GE, Cady KC, Koeppen K, Bhuju S, Stanton BA, O'Toole GA. 2015. Clustered regularly interspaced short palindromic repeat-dependent, biofilm-specific death of pseudomonas aeruginosa mediated by increased expression of phage-related genes. mBio 6:1–13.
6. Friedman L, Kolter R. 2004. Two genetic loci produce distinct carbohydrate-rich structural components of the. Journal of bacteriology 186:4457–4465.
7. Friedman L, Kolter R. 2004. Genes involved in matrix formation in *Pseudomonas aeruginosa* PA14 biofilms. Molecular Microbiology 51:675–690.
8. Colvin KM, Gordon VD, Murakami K, Borlee BR, Wozniak DJ, Wong GCL, Parsek MR. 2011. The Pel polysaccharide can serve a structural and protective role in the biofilm matrix of *Pseudomonas aeruginosa*. PLoS Pathogens 7:e1001264.
9. Kolter R, Bloemberg G V, O'Toole G, Lugtenberg BJ. 1997. Green fluorescent protein as a marker for *Pseudomonas* spp. Applied and Environmental Microbiology 63:4543.
10. Shanks RMQ, Caiazza NC, Hinsa SM, Toutain CM, O'Toole GA. 2006. *Saccharomyces cerevisiae*-based molecular tool kit for manipulation of genes from gram-negative bacteria. Applied and Environmental Microbiology 72:5027–5036.
11. Dahlstrom KM, Giglio KM, Sondermann H, O'Toolea GA. 2016. The inhibitory site of a diguanylate cyclase is a necessary element for interaction and signaling with an effector protein. Journal of Bacteriology 198:1595–1603.
12. Choi KH, Gaynor JB, White KG, Lopez C, Bosio CM, Karkhoff-Schweizer RAR,

Schweizer HP. 2005. A Tn7-based broad-range bacterial cloning and expression system. Nature Methods 2:443–448.

13. Choi KH, Schweizer HP. 2005. An improved method for rapid generation of unmarked Pseudomonas aeruginosa deletion mutants. BMC Microbiology 5:1–11.
14. Karimova G, Ullmann A, Ladant D. 2001. Protein-protein interaction between *Bacillus stearothermophilus* tyrosyl-tRNA synthetase subdomains revealed by a bacterial two-hybrid system. Journal of Molecular Microbiology and Biotechnology 3:73–82.
15. Whitfield GB, Marmont LS, Ostaszewski A, Rich JD, Whitney JC, Parsek MR, Harrison JJ, Howell PL. 2020. Pel polysaccharide biosynthesis requires an inner membrane complex comprised of PelD, PelE, PelF, and PelG. Journal of bacteriology 202:e00684-19.
16. Asikyan ML, Kus J V., Burrows LL. 2008. Novel proteins that modulate type IV pilus retraction dynamics in *Pseudomonas aeruginosa*. Journal of Bacteriology 190:7022–7034.
